# Surgical intervention versus conservative care in severe hypertensive pontine hemorrhage: a retrospective analysis of clinical outcomes

**DOI:** 10.3389/fsurg.2026.1853742

**Published:** 2026-06-15

**Authors:** Ming-Lu Li, Jian-Wang Zhang, Xiao-Qiong Su, Xu-Xiang Yu, Jun-Jiang Tong, Xin-Hua Tian, Gui-Jiang Dong, Zhong Liu

**Affiliations:** 1 Department of Neurosurgery, Zhongshan Hospital of Xiamen University, School of Medicine, Xiamen University, Xiamen, Fujian, China; 2 Department of Clinical Medicine, School of Medicine, Xiamen University, Xiamen, China; 3 Department of Neurosurgery, Anxi County Hospital, Quanzhou, China; 4 College of Plant Science and Technology, Hunan Biological and Electromechanical Polytechnic, Changsha, China

**Keywords:** craniotomy, hypertensive pontine hemorrhage, prognosis, stereotactic drainage, surgical treatment

## Abstract

**Background:**

Hypertensive pontine hemorrhage (HPH) carries high mortality and disability, with no standardized treatment guidelines. This study compared treatment efficacy for severe HPH and identified prognostic factors to guide clinical decisions.

**Methods:**

We retrospectively analyzed consecutive severe HPH patients (GCS 4-7, hematoma volume ≥ 5 mL, isolated pontine hemorrhage) treated at our center (2020-2024), stratified into pharmacological therapy, stereotactic drainage, and craniotomy evacuation groups. To eliminate treatment choice confounding bias, we performed 1:1 Propensity score matching (PSM) to generate an unbiased 112-patient cohort (56 pharmacological, 56 surgical), with standardized mean difference < 0.10 defining excellent intergroup balance. All core analyses were performed exclusively on this PSM-matched unbiased cohort. Inverse probability of treatment weighting (IPW) was used only as a sensitivity analysis to validate primary finding robustness. We assessed 30/90-day mortality and unfavorable functional outcomes (modified Rankin Scale ≥ 4), and recorded perioperative complications, ICU and total hospital stay for all patients. Multivariate logistic regression identified independent prognostic factors.

**Results:**

Post-PSM, all baseline covariates were well-balanced, eliminating treatment choice confounding bias. In the matched cohort, the surgical group had significantly lower 30/90-day mortality and 90-day unfavorable functional outcomes versus the pharmacological group (all *P* < 0.05); the 30-day unfavorable functional outcome rate showed no significant difference between the two groups (*P* = 0.087). These findings were fully validated by IPW sensitivity analysis. In surgical subgroup comparison, craniotomy achieved higher hematoma clearance but higher intracranial infection risk, while stereotactic drainage offered shorter ICU/hospital stays but higher rebleeding risk (all *P* < 0.05). Multivariate analysis identified older age, lower GCS score, larger hematoma volume, acute obstructive hydrocephalus, and massive hematoma morphology as independent adverse prognostic factors, with regular antihypertensive use as a protective factor.

**Conclusion:**

In this PSM-matched cohort, surgery correlated with significantly reduced 30/90-day mortality and 90-day unfavorable functional outcome risk, with stereotactic drainage and craniotomy showing divergent safety-efficacy profiles. Findings do not constitute formal treatment recommendations, but may inform individualized clinical decisions based on patient and hematoma characteristics. This retrospective single-center study cannot establish definitive causality; results should be interpreted cautiously, with large-scale multi-center prospective trials in unbiased populations needed to verify robustness.

## 1 Introduction

Hypertensive pontine hemorrhage (HPH), accounting for approximately 5%–10% of hypertensive intracerebral hemorrhages, represents one of the most critical subtypes in terms of disease severity (1–3), with an overall in-hospital mortality rate of 40%–60% and a long-term disability rate exceeding 80% in surviving patients (4–7). The pons, a core structure of the brainstem densely populated with nerve nuclei and ascending/descending tracts, is highly susceptible to severe neurological deficits following hemorrhage, including impaired consciousness, respiratory/circulatory dysfunction, and limb paralysis, leading to persistently high rates of mortality and disability (8, 9).

With the evolution of neuroimaging technology and advances in minimally invasive neurosurgical concepts, treatment options for HPH have become increasingly diverse. Currently, pharmacological therapy and surgical intervention are the primary clinical approaches, with no unified international clinical guidelines defining standardized treatment algorithms for severe cases to date (7, 10–13). Pharmacological treatment focuses on symptomatic and supportive care, primarily involving blood pressure control, intracranial pressure management, and prevention/treatment of complications (14). It is the first-line option for patients with a Glasgow Coma Scale (GCS) score ≥ 8, hematoma volume < 5 mL, or severe vital organ dysfunction precluding surgery (7, 14); however, its efficacy is often limited in cases with larger hematoma volume and significantly elevated intracranial pressure (15), with a reported unfavorable outcome rate (modified Rankin Scale, mRS ≥ 4) of over 80% in severe HPH treated pharmacologically (11, 16).

The core objectives of surgical intervention are to rapidly evacuate the hematoma, relieve brainstem compression, and lower intracranial pressure, thereby creating conditions for neurological recovery, with surgical candidates generally defined as patients with GCS 4-7, hematoma volume ≥ 5 mL, and no absolute surgical contraindications (3, 6, 12). Stereotactic hematoma puncture and drainage, with its advantages of precise targeting, minimal invasiveness, and operational convenience, has been widely adopted for intracranial hematoma evacuation and is reported to improve prognosis and reduce mortality (6, 11, 12, 14, 17). However, the specific rates of its complications, such as rebleeding and infection, were not reported in the original study. In contrast, other papers on intracranial hemorrhage have reported that the symptomatic rebleeding rate after puncture and drainage or minimally invasive surgery ranges from 2.4% to 5.8%, and the infection rate is approximately 0.8% (18, 19).

Craniotomy for hematoma evacuation allows direct visualization for hematoma removal and decompression but is associated with greater surgical trauma and demands high microsurgical technical skills from the surgeon, necessitating strict adherence to surgical indications (13, 20). It is a suitable approach for patients with severe brainstem compression, progressive neurological deterioration, or irregular/diffuse HPH (21). While the infection complications of craniotomy for HPH have not been reported, the infection rate for posterior fossa surgery, which is adjacent to the cerebrospinal fluid circulation pathway, ranges from 7.0% to 12% (7). Furthermore, HPH is frequently complicated by acute obstructive hydrocephalus, which occurs in 11%–32% of severe cases and exacerbates clinical deterioration (12, 14, 16). In such scenarios, the decision to perform external ventricular drainage (EVD) is crucial for patient prognosis, with timely drainage reported to reduce the 30-day mortality rate by approximately 20% in these patients (16). The choice of surgical approach significantly impacts outcomes. Although the telovelar and subtemporal transtentorial approaches are established routes to the pons, comparative analyses of their outcomes in well-defined cohorts of severe HPH (GCS < 8, hematoma volume ≥ 5 mL) are lacking, and the balance between surgical invasiveness, hematoma clearance efficacy, and complication risks remains poorly characterized in this specific population (13, 21).

This study retrospectively analyzed the clinical data of patients with HPH admitted to our hospital over the past five years, focusing on a strictly defined cohort of severe cases with unified inclusion criteria. It aimed to compare the efficacy differences between pharmacological therapy and various surgical strategies (stereotactic drainage and craniotomy evacuation) and to analyze independent prognostic influencing factors, thereby providing evidence-based support for formulating individualized clinical treatment plans and optimizing surgical approach selection for severe HPH.

## 2 Materials and methods

### 2.1 General data and observation indices

#### 2.1.1 General data

A total of 202 patients with severe hypertensive pontine hemorrhage were initially screened and admitted to the Departments of Neurology and Neurosurgery at our hospital between January 2020 and December 2024. After excluding 33 patients (traumatic hemorrhage: *n* = 11; secondary hemorrhage due to vascular malformation, aneurysm, or tumor: *n* = 5; severe vital organ dysfunction: *n* = 6; coagulation disorders: *n* = 4; brain death on admission: *n* = 3; incomplete follow-up data: *n* = 4), 169 patients were initially enrolled and stratified into the pharmacological therapy group (*n* = 58), stereotactic drainage subgroup (*n* = 72), and craniotomy evacuation subgroup (*n* = 39).

**Figure 1:**
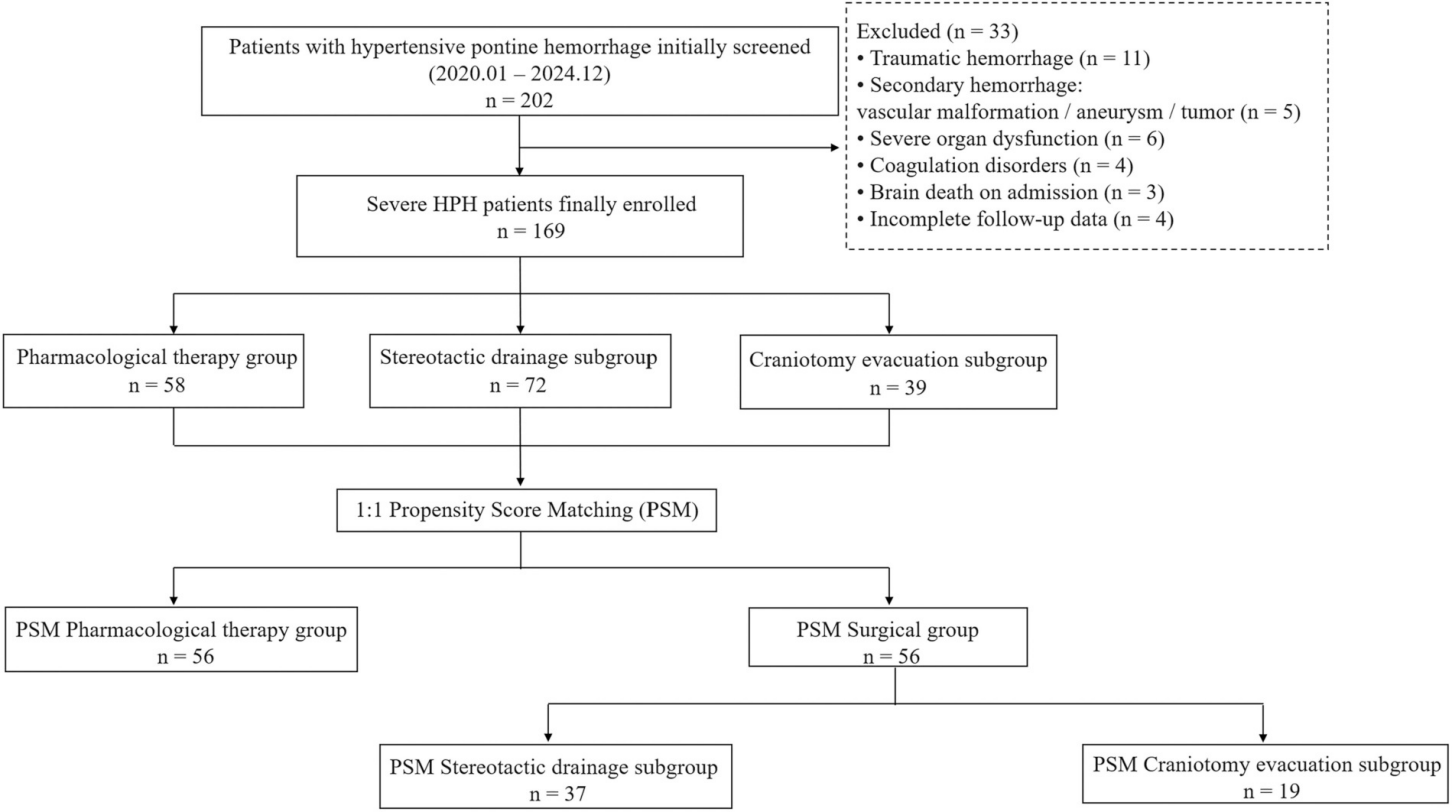
Flowchart of patient enrollment, screening, exclusion and grouping in the present study.

However, owing to non-randomized treatment allocation determined by family choice and surgeon recommendation, the initial 169-patient cohort carried significant selection bias. Therefore, all primary comparative analyses were performed exclusively in a 1:1 propensity score-matched (PSM) unbiased cohort, which included 56 patients in the pharmacological therapy group and 56 patients in the surgical group. Among the 56 matched surgical patients, 37 were treated with stereotactic drainage and 19 with craniotomy evacuation, consistent with the clinical distribution in the original cohort and balanced after PSM adjustment (Figure 1).

##### 2.1.1.1 Inclusion criteria

① Diagnosis of hypertensive intracerebral hemorrhage based on the American Heart Association/American Stroke Association (AHA/ASA) *Guidelines for the Management of Spontaneous Intracerebral Hemorrhage* (22), with hemorrhage foci located in the pons confirmed by cranial Computed Tomography (CT); ② Time from symptom onset to hospital admission ≤ 24 hours; ③ Age ≥ 18 years; ④ Preoperative GCS score of 4-7 with a hematoma volume ≥ 5 mL was operationally defined as severe HPH. ⑤ Complete clinical data.

##### 2.1.1.2 Criteria for treatment strategy selection

This inclusion criterion was designed based on clinical evidence and current guidelines: for patients with pontine hemorrhage presenting with an admission GCS score ≥ 8 or no coma, surgical intervention is generally not considered, and pharmacological treatment is the primary therapeutic option for this subgroup. In contrast, patients with an admission GCS score of 3 (deep coma) tend to die in a short period, with extremely poor therapeutic outcomes regardless of the treatment modality adopted (7, 22–25). In addition, the baseline characteristics of the three treatment groups were comparable with no statistical differences, which enabled a valid comparison of the therapeutic effects of the three different modalities under the premise of consistent baseline clinical conditions of the enrolled cases.

It is well known that the overall prognosis of such patients with severe hypertensive pontine hemorrhage remains poor. Notably, among the enrolled patients, only those complicated with acute obstructive hydrocephalus were recommended surgical intervention by our medical team; for all other enrolled patients, the choice of pharmacological treatment, stereotactic hematoma puncture and drainage, or craniotomy evacuation was mainly determined by the informed choice of the patients’ families after full and detailed communication of the risks, benefits and expected prognosis of each treatment modality by the multidisciplinary medical team.

##### 2.1.1.3 Exclusion criteria

① Traumatic pontine hemorrhage; ② Secondary pontine hemorrhage due to cerebrovascular malformation, intracranial aneurysm, brain tumor; ③ Concurrent severe dysfunction of vital organs (heart, liver, kidney); ④ Coagulation disorders; ⑤ Meeting brain death criteria upon admission; ⑥ Incomplete follow-up data.

Based on treatment strategy, patients were divided into a pharmacological therapy group (*n* = 58) and a surgical treatment group (*n* = 111). The surgical group was further subdivided into a stereotactic hematoma puncture and drainage subgroup (*n* = 72) and a craniotomy hematoma evacuation subgroup (*n* = 39). Within the surgical group, 42 patients complicated by acute obstructive hydrocephalus (evidenced by lateral and third ventricular dilation with cerebrospinal fluid pathway obstruction on cranial CT) underwent combined external ventricular drainage. In the craniotomy subgroup, the telovelar approach (suitable for dorsal hemorrhages) was used in 21 cases, and the subtemporal transtentorial approach (suitable for ventrolateral hemorrhages) in 18 cases. According to Wessels’ classification method (26), hematomas were categorized into three types: (1) dorsal; (2) ventral; (3) massive. In this study, massive hemorrhages accounted for over 60%, with dorsal and ventral hemorrhages having similar proportions.

#### 2.1.2 Observation indices

The following indices were observed: ① Baseline characteristics: Age, sex, body mass index (BMI), pre-morbid functional status assessed by the mRS score, blood lipid profile (total cholesterol, triglycerides, low-density lipoprotein cholesterol, high-density lipoprotein cholesterol), hemorrhage type (26) (ventral, dorsal, massive); ② Underlying diseases and condition assessment: History of hypertension, admission systolic/diastolic blood pressure, regularity of antihypertensive medication use, comorbidities (diabetes mellitus, coronary heart disease), admission blood glucose, admission GCS score, preoperative requirement for mechanical ventilation, preoperative requirement for tracheal intubation, time from onset to surgery; ③ Treatment-related indices: Preoperative hematoma volume, postoperative hematoma volume, hematoma clearance rate ([preoperative volume - postoperative volume]/preoperative volume  × 100%); ④ Prognostic indices: mRS score at 30 days and 90 days post-treatment; ⑤ Complications: Intracranial infection; postoperative rebleeding, defined as a radiologically confirmed increase in hematoma volume on follow-up cranial CT within 24 hours after surgery compared with the immediate postoperative CT, meeting at least one of the following criteria: (1) an increase in hematoma volume ≥ 33% relative to the immediate postoperative volume, or (2) the appearance of any new hemorrhagic foci remote from the surgical tract (27–29); lower extremity deep vein thrombosis; ⑥ Hospitalization indices: Length of stay in the intensive care unit (ICU), the overall length of hospital stay.

### 2.2 Imaging evaluation and hematoma volume calculation

All patients underwent preoperative and postoperative (24-hour) thin-slice cranial CT scans (slice thickness 0.6 mm). Hematoma volume (mL) was calculated using the open-source software 3D Slicer (version 5.2.2). The hematoma clearance rate (%) was calculated as: [Postoperative hematoma volume (mL) at 24 hours/Preoperative hematoma volume (mL)] × 100%.

### 2.3 Treatment methods

#### 2.3.1 Pharmacological treatment group

A standardized comprehensive neurocritical care protocol was implemented: ① Blood pressure management: Urapidil and nicardipine were the first-line titratable intravenous agents; sodium nitroprusside was only used rescue therapy in patients with refractory severe hypertension (SBP > 200 mmHg) unresponsive to first-line drugs. Only 7 patients (12%) in the pharmacological group received short-term sodium nitroprusside; all others were managed with urapidil/nicardipine.

Intravenous infusion of sodium nitroprusside or urapidil to maintain systolic blood pressure at 130 to 150 mmHg and diastolic blood pressure at 80 to 90 mmHg; for patients on regular antihypertensives, doses were adjusted dynamically based on blood pressure, while long-acting antihypertensive agents were initiated for those not previously on regular medication. ② Intracranial pressure control: Mannitol (125 mL, q6-8 h) combined with furosemide (20-40 mg, qd-bid) administered alternately, supplemented with glycerin fructose if necessary to reduce cerebral edema. ③ Neurocritical supportive care: Glycemic control (fasting blood glucose ≤ 7.8 mmol/L); statins for patients with dyslipidemia; maintenance of fluid, electrolyte, and acid-base balance; mechanical ventilation and tracheal intubation for patients with impaired consciousness or respiratory insufficiency; prevention of complications such as pulmonary infection and stress ulcer. All surgical patients received identical standardized neurocritical care as the pharmacological group, including BP control, glucose control, complication prevention, and supportive treatment, ensuring comparability between groups.

#### 2.3.2 Stereotactic hematoma puncture and drainage

Preoperative thin-slice CT scanning combined with intraoperative neuronavigation (BrainLAB, Germany) was performed to localize the hematoma and determine the scalp projection point corresponding to the maximal hematoma cross-section. Following general anesthesia, patients were placed in a lateral decubitus position, with the head fixed using a three-pin head holder. A linear incision approximately 2 cm in length was made at the preoperative navigation-guided scalp projection point. After standard skin disinfection and draping, the scalp was incised, followed by a burr hole and dural opening. A ventricular catheter was stereotactically placed using the neuronavigation system, with the target position aligned along the longitudinal axis of the hematoma. After catheter placement, the hematoma was aspirated using a syringe until significant resistance was encountered. Upon completion of aspiration, the catheter was left *in situ* within the hematoma cavity, and the scalp was closed in layers. The decision for concurrent external ventricular drainage was based on the presence of intraventricular hematoma extension and/or acute obstructive hydrocephalus. Postoperative cranial CT was routinely performed to verify catheter position. The drainage catheter was typically removed within 5–7 days postoperatively (12, 14).

Postoperatively, urokinase (20,000–50,000 IU) dissolved in 2 mL of normal saline was administered via the catheter into the hematoma cavity. The catheter was clamped for 2-3 hours before reopening for drainage, with a frequency of twice daily (17). The dosage of urokinase and duration of drainage were adjusted according to the drainage characteristics.

#### 2.3.3 Craniotomy and approach selection

The selection of surgical approaches for brainstem hemorrhage adhered to two fundamental principles: the “two-point method” for trajectory planning and entry through established anatomical safe zones (30). The two points defining the optimal trajectory were the center of the hematoma and the point on the brainstem surface nearest to the hematoma. The surgical approaches employed in this study included the subtemporal transtentorial approach and the telovelar approach (13, 21, 31, 32). The safe zones utilized on the pons comprised the ventrolateral supratrigeminal and peritrigeminal zones, and the dorsal supra-facial, infra-facial, and suprafacial midline zones. Notably, if the hematoma had ruptured through the pial surface of the brainstem, entry into the hematoma cavity was made directly via the rupture site.

##### 2.3.3.1 Suboccipital midline telovelar approach

Following successful anesthesia, the patient was positioned in a ¾ lateral prone position with an axillary roll placed. The head was fixed in a three-pin head holder, slightly flexed and tucked, to maximally open the atlanto-occipital joint and expose the craniocervical junction. The upper shoulder was retracted caudally to increase surgical working space. A midline suboccipital incision was made, extending from approximately 1–2 cm above the inion to the level of the C3 spinous process. The dissection proceeded along the midline avascular plane through the skin and nuchal ligament down to the occipital squama, followed by subperiosteal dissection. The posterior tubercle and posterior arch of C1 were exposed along the midline, and a subperiosteal dissection was continued to expose the posterior arch of C1 and the foramen magnum. Four burr holes were placed around the occipital squama, and a bone flap measuring approximately 5 × 6 cm was created using a craniotome. The foramen magnum and approximately 2 cm of the C1 posterior arch were further opened using rongeurs. The dura was opened in a Y-shaped fashion. An enlarged occipital sinus, if present, was ligated; otherwise, it was coagulated and divided. Under the microscope, the cerebellomedullary fissure was dissected to expose and open the tela choroidea and inferior medullary velum, revealing the floor of the fourth ventricle. Based on preoperative thin-slice CT, hematomas rupturing into the fourth ventricle were evacuated directly via the rupture site. If no rupture site was present, a cortical incision was made in a safe zone (supra-facial, infra-facial, or suprafacial midline) on the floor of the fourth ventricle to access and evacuate the hematoma. After evacuation, the cavity was irrigated, and hemostasis was achieved using flowable gelatin. Bipolar coagulation was generally avoided; if necessary, only low-power settings were used. The dura was closed watertight. The bone flap was either replaced or left out for decompression, followed by layered closure of the suboccipital muscles and skin (13).

##### 2.3.3.2 Subtemporal transtentorial approach

Following successful anesthesia, the patient was placed in a lateral position with an axillary roll. The head was fixed in a three-pin head holder and tilted approximately 20° downward to utilize gravity for temporal lobe retraction, exposing the middle fossa floor and tentorium. An lumbar drain was placed first. A preauricular curvilinear incision was made, starting 1 cm below the zygomatic arch and 1 cm anterior to the tragus, extending vertically to the superior temporal line, then curving posteriorly for approximately 2 cm. Upon skin incision, the lumbar drain was opened to slowly drain cerebrospinal fluid (approximately one drop every three seconds). The skin, subcutaneous tissue, and temporalis muscle were incised in layers to expose the temporal squama (two-thirds anterior and one-third posterior to the external auditory canal). A burr hole was placed at the level of the supramastoid crest, and a bone flap of approximately 4 × 4 cm was created with a craniotome, extending as low as possible along the middle fossa floor. Additional drilling of the temporal floor bone was performed to flatten the bony window to the level of the middle fossa floor. Any opened mastoid air cells were sealed with bone wax and irrigated with iodine solution. A horseshoe-shaped dural flap was opened with its base towards the middle fossa floor. Under the microscope, the temporal lobe was gently elevated, the arachnoid of the temporal base was opened, and the tentorium and trochlear nerve were exposed. Tentorial incision was started posterior to the trochlear nerve using a combination of coagulation and cutting in an alternating manner. In case of significant bleeding from a tentorial sinus, flowable gelatin was applied for hemostasis. The arachnoid of the ambient cistern was opened. Based on preoperative thin-slice CT, hematomas rupturing into the ambient cistern were evacuated directly via the rupture site. If no rupture site was present, a cortical incision was made in a ventrolateral safe zone (supratrigeminal or peritrigeminal) to access and evacuate the hematoma. The strategy for hematoma evacuation was similar to that described for the telovelar approach. The dura was closed watertight. The bone flap was replaced, followed by layered closure of the muscle and skin (21).

### 2.4 Prognostic assessment

Clinical outcomes were assessed using the mRS (11–14, 16, 17, 25). The specific mRS criteria were as follows: 0 – No symptoms; 1 – No significant disability despite symptoms, able to perform all usual duties and activities; 2 – Slight disability, unable to perform all previous activities but able to look after own affairs without assistance; 3 – Moderate disability, requiring some help but able to walk without assistance; 4 – Moderately severe disability, unable to walk without assistance and unable to attend to own bodily needs without assistance; 5 – Severe disability, bedridden, incontinent, requiring constant nursing care and attention; 6 – Death. An unfavorable outcome was defined as an mRS score ≥ 4.

### 2.5 Statistical analysis

Data were analyzed using SPSS software (version 26.0). Continuous variables were tested for normality. Normally distributed data are presented as mean ± standard deviation (x̅ ± s) and were compared between two groups using the independent samples *t*-test, among multiple groups using one-way ANOVA, with *post-hoc* pairwise comparisons performed using the LSD-*t* test. Non-normally distributed data are presented as median (interquartile range) [M (Q1, Q3)] and were compared between groups using the Wilcoxon rank-sum test or the Kruskal–Wallis H test. Categorical data are presented as counts (percentages) [n (%)] and were compared using the chi-square test or Fisher's exact test, as appropriate. Multivariate Logistic regression analysis was performed to identify independent preoperative prognostic factors for clinical outcomes (30-day mortality and 90-day unfavorable outcome).

To reduce selection bias and confounding by indication owing to non-randomized treatment allocation, 1:1 nearest-neighbor PSM was conducted with a caliper of 0.02. Baseline demographic and clinical variables were incorporated into the propensity score model. Standardized mean difference (SMD) was calculated to evaluate baseline comparability, an absolute SMD < 0.1 was considered as excellent balance (33), while SMD < 0.20 was considered acceptable balance (34). Inverse probability of treatment weighting (IPW) was further performed as a sensitivity analysis to verify the robustness of the primary results.

## 3 Results

### 3.1 Baseline characteristics in the PSM-matched cohort

After 1:1 propensity score matching, 56 patients in the pharmacological therapy group and 56 patients in the surgical group were included in the primary analysis. As detailed in Table 1, all baseline demographic, clinical, laboratory, and imaging variables were well balanced between the two groups after matching, with most standardized mean differences (SMDs) below 0.10. Specifically, four covariates (age, admission diastolic blood pressure, high-density lipoprotein cholesterol, and preoperative hematoma volume) exhibited SMDs slightly above 0.10 (ranging from 0.11 to 0.16), which were still within the acceptable range of SMD < 0.20 (per our predefined balance criteria). No meaningful differences were observed in other baseline characteristics, including sex, body mass index, history of hypertension, admission systolic blood pressure, blood glucose, comorbidities, admission GCS score, acute obstructive hydrocephalus, or hemorrhage type. All patients had a premorbid mRS score of 0–1, indicating independent living status with no preexisting neurological deficits. This unbiased, well-balanced cohort provides a valid foundation for subsequent comparative analyses of treatment efficacy.

**Table 1:** Baseline characteristics of pharmacological vs. surgical groups before and after 1:1 propensity score matching (PSM).

Variable	Before matching			After matching		
	Pharmacological (*n* = 58)	Surgical (*n* = 111)	SMD	Pharmacological (*n* = 56)	Surgical (*n* = 56)	SMD
Demographic characteristics						
Age, years	54 ± 9	55 ± 8	0.12	54 ± 9	55 ± 8	0.11
Sex, male, *n* (%)	43 (74)	83 (75)	0.02	41 (73)	42 (75)	0.04
BMI, kg/m^2^	24 ± 3	24 ± 3	0.00	24 ± 3	24 ± 3	0.00
Clinical parameters						
Hypertension history	55 (95)	106 (95)	0.01	53 (95)	53 (95)	0.00
Regular antihypertensive use	6 (10)	11 (10)	0.01	6 (11)	5 (9)	0.06
Admission SBP, mmHg	179 ± 20	183 ± 21	0.20	180 ± 20	182 ± 21	0.10
Admission DBP, mmHg	102 ± 13	106 ± 13	0.31	103 ± 13	105 ± 13	0.15
Admission blood glucose, mmol/L	7.8 ± 2.1	8.2 ± 2.4	0.18	7.9 ± 2.1	8.1 ± 2.3	0.09
Diabetes mellitus	12 (21)	22 (20)	0.03	11 (20)	11 (20)	0.00
Coronary heart disease	9 (16)	17 (15)	0.01	9 (16)	8 (14)	0.05
Pre-morbid mRS score (0–1)	58 (100)	111 (100)	–	56 (100)	56 (100)	–
Laboratory values						
Total cholesterol, mmol/L	5.2 ± 1.1	5.2 ± 1.1	0.00	5.2 ± 1.1	5.2 ± 1.1	0.00
Triglycerides, mmol/L	1.8 ± 0.7	1.8 ± 0.7	0.00	1.8 ± 0.7	1.8 ± 0.7	0.00
LDL-C, mmol/L	3.3 ± 0.9	3.3 ± 0.9	0.00	3.3 ± 0.9	3.3 ± 0.9	0.00
HDL-C, mmol/L	1.2 ± 0.3	1.1 ± 0.3	0.33	1.2 ± 0.3	1.1 ± 0.3	0.16
Preoperative status & acute complications						
Mechanical ventilation	28 (48)	78 (70)	0.46	38 (68)	37 (66)	0.04
Tracheal intubation	25 (43)	71 (64)	0.43	36 (64)	35 (63)	0.02
Admission GCS score	6.5 ± 1.2	6.2 ± 1.1	0.26	6.4 ± 1.2	6.3 ± 1.1	0.09
Pre-op hematoma volume, mL	7.2 ± 1.5	7.6 ± 1.6	0.26	7.3 ± 1.5	7.5 ± 1.6	0.13
Acute obstructive hydrocephalus	11 (19)	21 (19)	0.00	10 (18)	11 (20)	0.05
Hemorrhage type						
Ventral	10 (17)	19 (17)	0.00	9 (16)	10 (18)	0.05
Dorsal	11 (19)	21 (19)	0.00	11 (20)	10 (18)	0.05
Massive	37 (64)	71 (64)	0.00	36 (64)	36 (64)	0.00

Data are presented as mean ± standard deviation for continuous variables or *n* (%) for categorical variables. SMD, standardized mean difference; SMD < 0.20 indicates excellent intergroup balance.

Notably, the absolute differences for these covariates with SMDs between 0.11 and 0.16 were clinically negligible (e.g., age difference of 1 year, admission DBP difference of 2 mmHg, preoperative hematoma volume difference of 0.2 mL). Thus, these imbalances were not considered to bias the treatment effect estimates.

### 3.2 Treatment-related parameters in the PSM-matched cohort

Treatment-related parameters were compared in the PSM-matched cohort, including ICU length of stay, overall hospital stay, and surgical-specific indicators. The results are presented in Table 2.

**Table 2:** Comparison of treatment-related parameters among the three treatment groups in the matched cohort.

Parameter	Pharmacological therapy (*n* = 56)	Stereotactic drainage (*n* = 37)	Craniotomy evacuation (*n* = 19)	*P*
Postoperative hematoma volume, mL	–	2.7 ± 1.0	0.8 ± 0.4	<0.001
Hematoma clearance rate, %	–	75 ± 13	90 ± 12	<0.001
ICU length of stay, days	11 ± 3	8 ± 3	10 ± 3	<0.001
Overall hospital stay, days	28 ± 6	19 ± 4	22 ± 5	<0.001

Pharmacological therapy group did not receive surgical intervention, so postoperative hematoma volume and hematoma clearance rate were not measured. Pairwise comparisons: pharmacological vs. stereotactic: *P* < 0.001; pharmacological vs. craniotomy: *P* < 0.001; stereotactic vs. craniotomy: < 0.001. Data are presented as mean ± standard deviation. ICU, intensive care unit.

#### 3.2.1 ICU length of stay

In the PSM-matched cohort, the pharmacological therapy group had a significantly longer ICU length of stay (11 ± 3 days) than both stereotactic drainage (8 ± 3 days) and craniotomy evacuation (10 ± 3 days, all *P* < 0.05). The stereotactic drainage subgroup also showed a significantly shorter ICU stay than the craniotomy subgroup.

#### 3.2.2 Overall hospital stay

The pharmacological therapy group had the longest overall hospital stay (28 ± 6 days), followed by the craniotomy evacuation subgroup (22 ± 5 days), and the stereotactic drainage subgroup had the shortest duration (19 ± 4 days). All pairwise comparisons reached statistical significance (all *P* < 0.05).

#### 3.2.3 Surgical-specific parameters

In the surgical subgroups of the matched cohort, the stereotactic drainage subgroup had a larger postoperative residual hematoma volume (2.7 ± 1.0 mL) than the craniotomy evacuation subgroup (0.8 ± 0.4 mL, *P* < 0.001). Correspondingly, the craniotomy evacuation subgroup achieved a significantly higher hematoma clearance rate (90 ± 12%) than the stereotactic drainage subgroup (75 ± 13%, *P* < 0.001).

### 3.3 Thirty-day and ninety-day mortality and unfavorable outcomes in the PSM-matched cohort

We completed 90-day follow-up for all 112 patients in the PSM-matched cohort (56 in the pharmacological therapy group, 37 in the stereotactic drainage subgroup, and 19 in the craniotomy evacuation subgroup), achieving a 100% follow-up rate to ensure grouping consistency and validity of long-term prognostic analysis. All outcomes were assessed using the modified Rankin Scale (mRS), with mortality defined as mRS = 6 and unfavorable functional outcome defined as mRS ≥ 4. The primary analysis results are presented in Table 3, and the sensitivity analysis results are presented in Table 4.

**Table 3:** Thirty-day and ninety-day mortality and unfavorable outcomes in the PSM-matched cohort.

Outcome index	Pharmacological therapy (*n* = 56)	Stereotactic drainage (*n* = 37)	Craniotomy evacuation (*n* = 19)	Pharmacological vs. surgical (*P*)	Stereotactic vs. craniotomy (*P*)
30-day mortality (mRS = 6)	30 (54%)	16 (43%)	7 (37%)	<0.05	>0.05
90-day mortality (mRS = 6)	35 (63%)	18 (49%)	10 (53%)	<0.05	>0.05
30-day unfavorable Outcome (mRS ≥ 4)	46 (82%)	26 (70%)	14 (74%)	0.087	>0.05
90-day unfavorable Outcome (mRS ≥ 4)	49 (88%)	28 (76%)	15 (79%)	<0.05	>0.05

Data are presented as *n* (%). mRS, modified rankin scale. Mortality was defined as mRS = 6; unfavorable functional outcome was defined as mRS ≥ 4. No significant difference was observed between the stereotactic drainage subgroup and craniotomy evacuation subgroup for all 30-day and 90-day outcomes (*P* > 0.05).

**Table 4:** IPW sensitivity analysis results for clinical outcomes in the full cohort (*n* = 169).

Outcome index	Surgical vs. pharmacological therapy	
	OR (95% CI)	*P*
30-day mortality (mRS = 6)	0.57 (0.32–0.99)	0.047
90-day mortality (mRS = 6)	0.55 (0.31–0.97)	0.039
30-day unfavorable outcome (mRS ≥ 4)	0.60 (0.33–1.08)	0.087
90-day unfavorable outcome (mRS ≥ 4)	0.52 (0.28–0.94)	0.030

IPW, inverse probability of treatment weighting; OR, odds ratio; CI, confidence interval; mRS, modified rankin scale. The propensity score model for IPW was fully consistent with the PSM model, incorporating all baseline covariates.

#### 3.3.1 Mortality

In the PSM-matched cohort, the overall 30-day mortality rate was 47% (53/112), and the 90-day mortality rate was 56% (63/112), consistent with the delayed mortality pattern of severe HPH. Stratified analysis revealed that the pharmacological therapy group had a significantly higher 30-day mortality rate (54%, 30/56) than the pooled surgical group (41%, 23/56; *P* < 0.05). This survival benefit persisted at 90 days: the pharmacological therapy group had a mortality rate of 63% (35/56), which remained significantly higher than the pooled surgical group (50%, 28/56; *P* < 0.05). Within surgical subgroups, 30-day mortality was 43% (16/37) in the stereotactic drainage subgroup and 37% (7/19) in the craniotomy evacuation subgroup, with no significant difference between the two approaches (*P* > 0.05). At 90 days, mortality rates were 49% (18/37) and 53% (10/19) in the stereotactic drainage and craniotomy evacuation subgroups, respectively, with no statistically significant difference (*P* > 0.05) (Table 3).

#### 3.3.2 Unfavorable outcomes (mRS ≥ 4)

In the PSM-matched cohort, the overall 30-day unfavorable outcome rate was 77% (86/112), and the 90-day unfavorable outcome rate was 82% (92/112). Stratified analysis showed no significant difference in 30-day unfavorable outcome rate between the pharmacological therapy group (82%, 46/56) and the pooled surgical group (71%, 40/56) (*P* = 0.087). At 90 days, the pharmacological therapy group still had a significantly higher unfavorable outcome rate (88%, 49/56) than the pooled surgical group (77%, 43/56; *P* < 0.05). Within surgical subgroups, 30-day unfavorable outcome rates were 70% (26/37) in the stereotactic drainage subgroup and 74% (14/19) in the craniotomy evacuation subgroup, with no significant difference (*P* > 0.05). At 90 days, these rates were 76% (28/37) and 79% (15/19), respectively, with no statistically significant difference between the two surgical approaches (*P* > 0.05) (Table 3).

#### 3.3.3 IPW sensitivity analysis

To further validate the robustness of the primary findings derived from the PSM-matched cohort and minimize residual confounding bias in the full initial cohort, we performed IPW analysis based on the 169-patient full cohort. The propensity score model for IPW was fully consistent with the PSM model, incorporating all baseline demographic, clinical, laboratory, and imaging variables. Stabilized inverse probability weights were calculated for each patient, and weighted logistic regression was used to evaluate the independent association between surgical intervention and clinical outcomes.

The IPW analysis fully replicated the primary findings from the PSM-matched cohort: surgical intervention was independently associated with a significantly reduced risk of 30-day mortality (OR = 0.57, 95% CI: 0.32–0.99, *P* = 0.047), 90-day mortality (OR = 0.55, 95% CI: 0.31–0.97, *P* = 0.039), and 90-day unfavorable functional outcome (OR = 0.52, 95% CI: 0.28–0.94, *P* = 0.030). No significant difference was observed in 30-day unfavorable functional outcome between groups (OR = 0.60, 95% CI: 0.33–1.08, *P* = 0.087), which was also fully consistent with the PSM-matched results. These consistent findings across two gold-standard confounding control methods further strengthen the reliability of our conclusion that surgical intervention improves survival and long-term functional outcomes in patients with severe HPH (Table 4).

### 3.4 Clinical complications in the PSM-matched cohort

Clinical complication rates were compared in the PSM-matched cohort using chi-square tests or Fisher's exact test, as appropriate. The overall incidence of intracranial infection in the matched cohort was 4.5% (5/112), with significant differences among the three treatment groups (*P* = 0.028). Specifically, the incidence of intracranial infection was significantly higher in the craniotomy evacuation subgroup (16%, 3/19) compared to both the stereotactic drainage subgroup (5.4%, 2/37; *P* = 0.028) and the pharmacological therapy group (0.0%, 0/56). Postoperative rebleeding occurred exclusively in the stereotactic drainage subgroup, with an overall incidence of 1.8% (2/112) in the matched cohort. The rebleeding rate in the stereotactic drainage subgroup was 5.4% (2/37), which was significantly higher than the 0.0% rate observed in the craniotomy evacuation subgroup (*P* = 0.028). The incidence of lower extremity deep vein thrombosis (DVT) did not differ significantly among the three treatment groups in the matched cohort (*P* = 0.271), with overall rates of 13% (7/56) in the pharmacological therapy group, 16% (6/37) in the stereotactic drainage subgroup, and 21% (4/19) in the craniotomy evacuation subgroup. The results are presented in Table 5.

**Table 5:** Clinical complications among treatment groups in the PSM-matched cohort.

Complication	Pharmacological therapy (*n* = 56)	Stereotactic drainage (*n* = 37)	Craniotomy evacuation (*n* = 19)	Overall P (three groups)	Pairwise comparison (P)
Intracranial infection	0 (0.0%)	2 (5.4%)	3 (16%)	0.028	Craniotomy vs. Pharmacological: <0.05; Craniotomy vs. Stereotactic: <0.05
Postoperative rebleeding	—	2 (5.4%)	0 (0.0%)	0.028	Stereotactic vs. Craniotomy: <0.05
Lower extremity DVT	7 (13%)	6 (16%)	4 (21%)	0.271	—

### 3.5 Multivariate analysis of prognostic factors in the PSM-matched cohort

We performed multivariate Logistic regression analyses to identify independent preoperative prognostic factors for 30-day mortality and 90-day unfavorable outcome (mRS ≥ 4) in the PSM-matched unbiased cohort of 112 patients with severe hypertensive pontine hemorrhage. The model exclusively included preoperative/baseline variables available at the time of clinical treatment decision-making: age, gender, admission GCS score, hematoma volume, hemorrhage type, regular antihypertensive use, comorbidities (diabetes mellitus, coronary heart disease), and presence of acute obstructive hydrocephalus at admission. Results are presented in Table 6 as odds ratios (OR) with 95% confidence intervals (95% CI) and *P* values.

**Table 6:** Independent preoperative prognostic factors for 30-day and 90-day outcomes in the PSM-matched cohort (multivariate logistic regression analysis).

Variable	30-day mortality (OR, 95% CI, *P*)	90-day unfavorable outcome (mRS ≥ 4) (OR, 95% CI, *P*)
Age (per 1 year increase)	1.05 (1.01–1.10, *P* = 0.015)	1.04 (1.00–1.09, *P* = 0.042)
Male gender (vs. female)	1.23 (0.67–2.25, *P* = 0.502)	1.19 (0.64–2.20, *P* = 0.581)
Admission GCS score (per 1 point increase)	0.71 (0.62–0.81, *P* < 0.001)	0.70 (0.60–0.80, *P* < 0.001)
Hematoma volume (per 1 mL increase)	1.13 (1.04–1.22, *P* = 0.004)	1.10 (1.02–1.19, *P* = 0.013)
Massive hematoma morphology (vs. other)	1.70 (0.82–3.52, *P* = 0.150)	2.90 (1.32–6.35, *P* = 0.008)
Ventral hematoma location (vs. dorsal)	0.88 (0.46–1.70, *P* = 0.715)	0.92 (0.48–1.75, *P* = 0.802)
Regular antihypertensive use (vs. no)	0.57 (0.31–1.06, *P* = 0.075)	0.54 (0.29–0.98, *P* = 0.045)
Diabetes mellitus (vs. no)	1.32 (0.64–2.70, *P* = 0.450)	1.28 (0.62–2.65, *P* = 0.502)
Coronary heart disease (vs. no)	1.16 (0.53–2.55, *P* = 0.720)	1.13 (0.50–2.55, *P* = 0.775)
Acute obstructive hydrocephalus (vs. no)	3.21 (1.40–7.35, *P* = 0.006)	2.98 (1.30–6.85, *P* = 0.010)

Only preoperative/baseline variables were included in the model; postoperative variables (hematoma clearance rate, postoperative rebleeding) were excluded to eliminate post-treatment bias. OR, odds ratio; 95% CI, 95% confidence interval; GCS, Glasgow Coma Scale; mRS, modified rankin scale. Statistically significant factors (*P* < 0.05) are the core independent prognostic factors for clinical reference.

For 30-day mortality in the matched cohort, independent risk factors included older age (OR = 1.05, 95% CI: 1.01–1.10, *P* = 0.015), lower admission GCS score (OR = 0.71, 95% CI: 0.62–0.81, *P* < 0.001), larger hematoma volume (OR = 1.13, 95% CI: 1.04–1.22, *P* = 0.004), and presence of acute obstructive hydrocephalus (OR = 3.21, 95% CI: 1.40–7.35, *P* = 0.006).

For 90-day unfavorable outcomes in the matched cohort, independent predictors were largely consistent with those for 30-day mortality, with two additional significant factors: massive hematoma morphology (OR = 2.90, 95% CI: 1.32–6.35, *P* = 0.008) and regular preoperative antihypertensive use (OR = 0.54, 95% CI: 0.29–0.98, *P* = 0.045). This model focused exclusively on preoperative variables that can directly inform clinical decision-making, providing actionable evidence for individualized prognostic assessment in patients with severe HPH.

### 3.6 Analysis of postoperative variables and their association with clinical outcomes in the PSM-matched surgical cohort

To clarify the role of postoperative variables as intermediate outcomes of surgical strategies, we performed a separate subgroup analysis exclusively in the surgical patients of the PSM-matched cohort (stereotactic drainage + craniotomy evacuation, *n* = 56), excluding the pharmacological conservative group which did not undergo surgical intervention (Table 7).

**Table 7:** Association of postoperative variables with 30-day and 90-day clinical outcomes in the PSM-matched surgical subgroups, stratified by surgical method.

Postoperative variable & stratification	30-day mortality, *n* (%)	90-day unfavorable outcome (mRS ≥ 4), *n* (%)	*P*
Hematoma clearance rate			
Overall (surgical cohort, *n* = 56)			
Complete clearance ≥ 80% (*n* = 36)	11 (31%)	24 (67%)	0.032
Incomplete clearance < 80% (*n* = 20)	12 (60%)	18 (90%)	0.025
By surgical method			
Stereotactic drainage (*n* = 37)			
Complete clearance ≥ 80% (*n* = 20)	6 (30%)	15 (75%)	0.045
Incomplete clearance < 80% (*n* = 17)	10 (59%)	16 (94%)	0.038
Craniotomy evacuation (*n* = 19)			
Complete clearance ≥ 80% (*n* = 16)	5 (31%)	9 (56%)	0.092
Incomplete clearance < 80% (*n* = 3)	2 (67%)	2 (100%)	0.078
Postoperative rebleeding			
Overall (surgical cohort, *n* = 56)			
With rebleeding (*n* = 2)	2 (100%)	2 (100%)	0.002
Without rebleeding (*n* = 54)	21 (39%)	41 (76%)	0.028
By surgical method			
Stereotactic drainage (*n* = 37)			
With rebleeding (*n* = 2)	2 (100%)	2 (100%)	0.005
Without rebleeding (*n* = 35)	14 (40%)	26 (74%)	0.032
Craniotomy evacuation (*n* = 19)			
With rebleeding (*n* = 0)	—	—	—
Without rebleeding (*n* = 19)	7 (37%)	15 (79%)	—

Analysis was restricted to the surgical subgroups of the PSM-matched cohort (stereotactic drainage + craniotomy evacuation, *n* = 56); the pharmacological therapy group was excluded due to no surgical intervention. *χ*^2^ test was used for statistical comparison; *P* < 0.05 was considered statistically significant. mRS, modified rankin scale.

#### 3.6.1 Hematoma clearance rate

Surgical patients in the matched cohort were stratified into complete clearance (clearance rate ≥ 80%) and incomplete clearance (clearance rate < 80%) groups. Among the 56 matched surgical patients, 36 (64%) achieved complete hematoma clearance, while 20 (36%) had incomplete clearance. When stratified by surgical method, the craniotomy evacuation subgroup achieved a significantly higher rate of complete hematoma clearance (84%, 16/19) compared to the stereotactic drainage subgroup (54%, 20/37; *P* = 0.003). Statistical analysis revealed that patients with complete hematoma clearance had significantly better 30-day and 90-day outcomes compared to those with incomplete clearance, with lower 30-day mortality (31% vs. 60%, *P* = 0.032) and lower 90-day unfavorable outcome rates (67% vs. 90%, *P* = 0.025), confirming that complete hematoma clearance is associated with improved long-term neurological recovery and reduced mortality in surgical patients. This association between complete clearance and improved outcomes was consistently observed in both surgical subgroups.

#### 3.6.2 Postoperative rebleeding

Postoperative rebleeding occurred in 2 (3.6%) of the 56 matched surgical patients, all in the stereotactic drainage subgroup (2/37, 5.4%), with no rebleeding events observed in the craniotomy evacuation subgroup, consistent with the complication analysis results. Comparison of outcomes between patients with and without postoperative rebleeding showed that rebleeding was a strong risk factor for poor prognosis, with 100% 30-day mortality (2/2) and 100% 90-day unfavorable outcome rate in patients with rebleeding, compared to 39% and 76% in those without (*P* = 0.002 and *P* = 0.028, respectively), highlighting the critical need for strict perioperative hemostatic management to minimize this life-threatening complication.

### 3.7 Case illustrations

#### 3.7.1 Case 1: Stereotactic hematoma puncture and drainage with urokinase instillation for HPH

**Figure 2:**
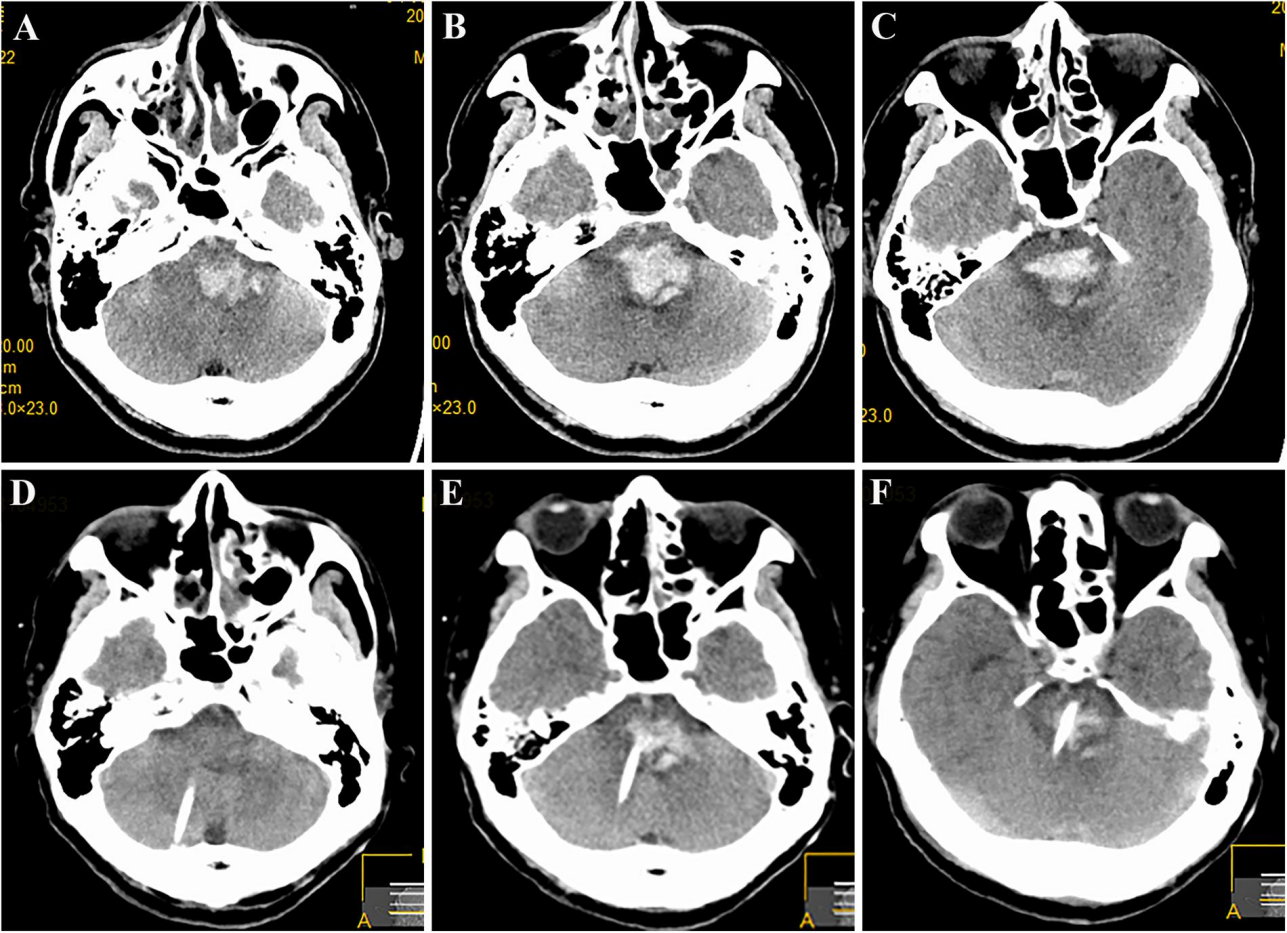
Stereotactic hematoma puncture and drainage with urokinase instillation for hypertensive pontine hemorrhage. (**A-C**) Computed tomography (CT) of the head demonstrated a pontine hematoma measuring approximately 9 mL in volume, without evidence of hydrocephalus. Emergency stereotactic puncture and drainage of the hematoma were performed. Intraoperatively, approximately 3 mL of altered blood was aspirated. Postoperatively, urokinase (30,000 IU in 2 mL normal saline) was instilled into the hematoma cavity daily via the catheter. (**D-F**) Follow-up head CT on postoperative day 3 revealed reduction of the hematoma volume to 2 mL.

A 52-year-old male presented with sudden-onset impaired consciousness lasting 3 hours. He had a 10-year history of hypertension and was not on regular antihypertensive therapy. On admission, his blood pressure was 185/108 mmHg. Neurological examination revealed shallow coma, bilateral pupils equal and round with sluggish light reflexes, muscle strength of grade 1 in all extremities, positive pathological signs, and mildly irregular respiratory rhythm. The GCS score was 6 (E1V2M3). CT of the head demonstrated a pontine hematoma measuring approximately 9 mL in volume, without evidence of hydrocephalus. The patient met the study's severe hypertensive pontine hemorrhage criteria (GCS = 6, hematoma volume 9 mL), and the family opted for stereotactic hematoma puncture and drainage after informed consent. Emergency stereotactic puncture and drainage of the hematoma were performed. Intraoperatively, approximately 3 mL of altered blood was aspirated. Postoperatively, urokinase (30,000 IU in 2 mL normal saline) was instilled into the hematoma cavity daily via the catheter, which was clamped for 3 hours before reopening for drainage; this regimen was repeated twice daily. Follow-up head CT on postoperative day 3 revealed reduction of the hematoma volume to 2 mL, corresponding to a clearance rate of approximately 73%. The drainage catheter was subsequently removed. By postoperative week 2, the patient's consciousness had improved to drowsiness, with a GCS score of 12 and muscle strength recovering to grade 3 in all limbs. At 30 days after treatment, the mRS score was 4, indicating moderately severe disability; the patient could sit with assistance. No complications such as intracranial infection or rebleeding were observed (Figure 2).

#### 3.7.2 Case 2: Telovelar approach for evacuation of a dorsal pontine hematoma with fourth ventricular rupture

**Figure 3:**
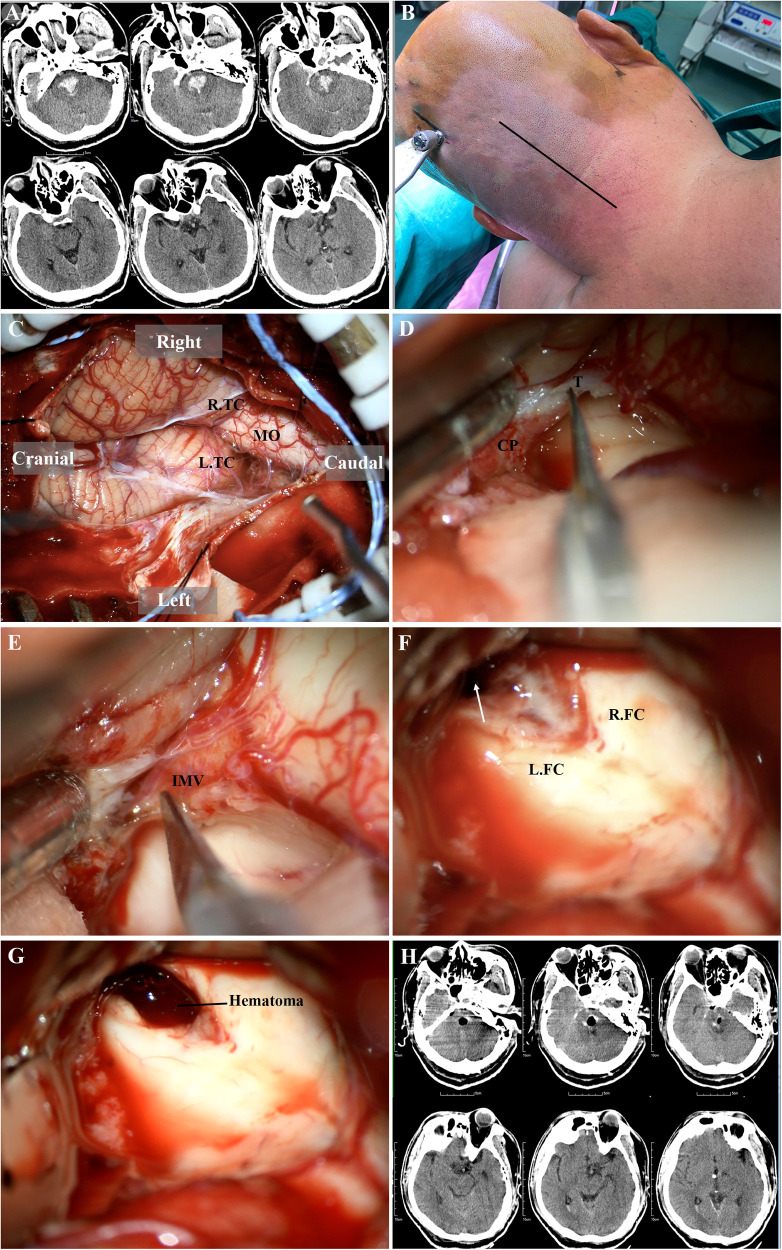
Telovelar approach for pontine hematoma evacuation. **(A)** Cranial CT shows pontine hemorrhage. **(B)** During surgery, the patient was placed in the left ¾ lateral prone position, with a posterior midline vertical incision made to perform the telovelar approach for hematoma evacuation. **(C)** The occipital squama and C1 posterior arch were removed, and the dura mater was incised in a Y-shape to expose the medulla oblongata, bilateral cerebellar tonsils, and cerebellar hemispheres. **(D,E)** The cerebellomedullary fissure was sharply dissected; the tela choroidea **(D)** was separated and the inferior medullary velum **(E)** was incised to expose the floor of the fourth ventricle. **(F)** The rupture of the pontine hematoma at the floor of the fourth ventricle was visible (Arrow). **(G)** Access was gained through the hematoma rupture to evacuate the hematoma. **(H)** Postoperative recheck CT indicated satisfactory hematoma evacuation. L, Left; R, Right; MO, Medulla oblongata; TC, Tonsil of cerebellum; CP, Choroid plexus; IMV, Inferior medullary velum; FC, Facial colliculus.

A 48-year-old female was admitted due to sudden headache accompanied by confusion for 6 hours. She had an 8-year history of hypertension and was regularly taking nifedipine sustained-release tablets. Admission blood pressure was 172/98 mmHg. Examination showed shallow coma, bilateral pupils 2 mm in diameter with preserved light reflexes, right-sided muscle strength grade 2, left-sided grade 3, neck stiffness, and two episodes of projectile vomiting. The GCS score was 7 (E2V3M2). Head CT identified a dorsal pontine hematoma measuring approximately 7 mL, with rupture into the fourth ventricle. The patient met the study's severe hypertensive pontine hemorrhage criteria (GCS = 7, hematoma volume 7 mL), the family opted for surgical treatment after informed consent, and the telovelar approach was selected according to the dorsal location of the hematoma and the brainstem safe entry zone principles. Emergency surgical evacuation via the telovelar approach was undertaken. Under general anesthesia in the prone position, a midline suboccipital craniotomy was performed to expose the foramen magnum and posterior arch of the atlas. Following dural opening, the fourth ventricle was visualized under microsurgical guidance. The hematoma in the dorsal pons and associated intraventricular blood were evacuated through the natural telovelar cleft. Postoperative management included routine measures for infection prophylaxis and intracranial pressure control. A head CT scan on postoperative day 1 indicated a hematoma clearance rate of approximately 92%. The external ventricular drain was removed one week postoperatively, and the patient's consciousness improved to a drowsy state. At 30 days post-treatment, the mRS score was 4. On postoperative day 5, the patient developed low-grade fever; cerebrospinal fluid analysis suggested mild inflammation, which resolved following targeted antibiotic therapy (Figure 3).

#### 3.7.3 Case 3: Subtemporal transtentorial approach for evacuation of a lateral HPH

**Figure 4:**
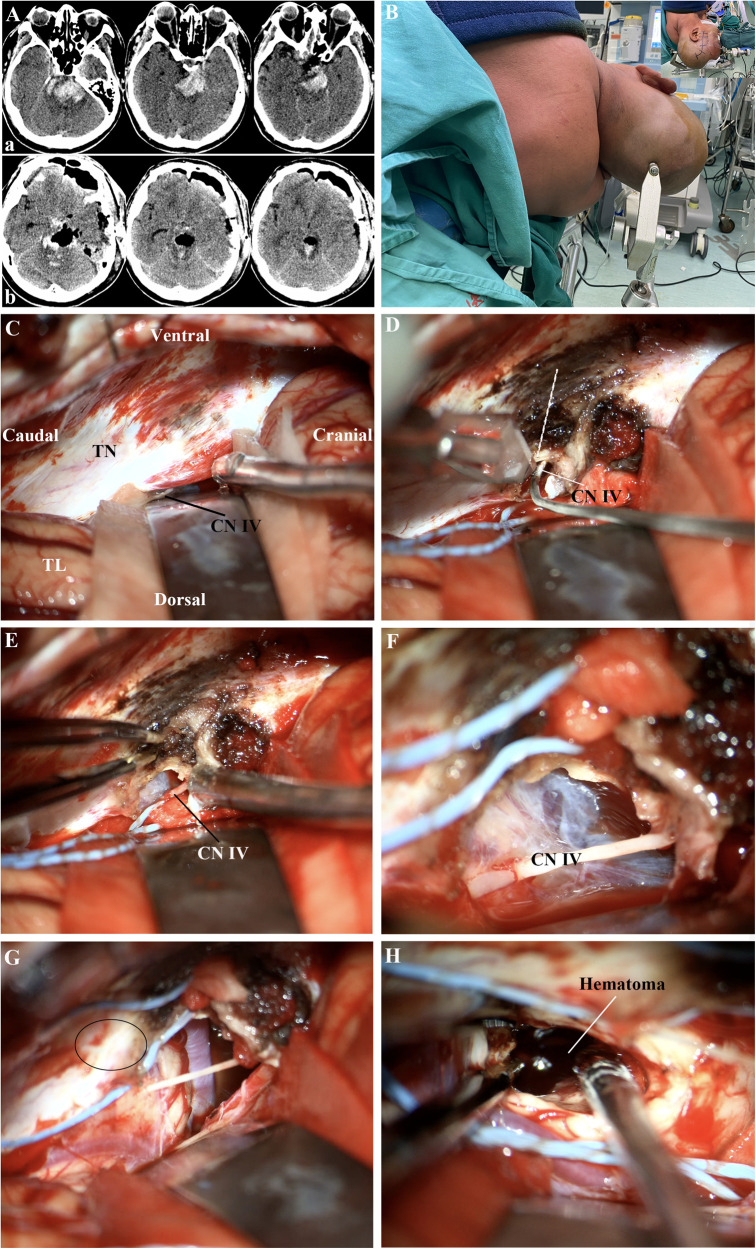
Left subtemporal trans-tentorial approach for pontine hematoma evacuation. **(A)** Cranial CT scans reveal pontine hemorrhage (a) and postoperative follow-up imaging (b) **(B)** The patient was placed in the left lateral position with the zygomatic arch maintained horizontal; the head was flexed downward at approximately 20°, and a preauricular curved incision was made (Upper right inset). **(C)** The arachnoid membrane at the temporal base was dissected to release cerebrospinal fluid, and gravity-assisted retraction of the temporal lobe was performed to expose the tentorium cerebelli and trochlear nerve. **(D,E)** The tentorium cerebelli was incised posterior to the trochlear nerve (Dashed line), with electrocoagulation applied intraoperatively during the incision. **(F)** After tentorial incision, the trochlear nerve was confirmed to be completely preserved, and the pons was fully visualized. **(G,H)** The surgical access was achieved via the supratrigeminal zone (Solid black circle) **(G)**, followed by thorough hematoma evacuation **(H)** TC, Tentorium cerebelli; CN Ⅳ, Trochlear nerve.

A 55-year-old male presented with acute left hemiplegia and consciousness disturbance of 4 hours’ duration. His medical history included hypertension for 12 years, without consistent medication adherence. Admission blood pressure was 190/110 mmHg. Neurological assessment revealed shallow coma, left pupil diameter 3 mm with sluggish light reflex, right pupil 2 mm with normal reactivity, left limb muscle strength grade 1, right limb grade 2, and positive pathological signs. The GCS score was 6 (E1V2M3). Head CT demonstrated a pontine hematoma with a laterally situated rupture point, measuring approximately 9 mL. The patient met the study's severe hypertensive pontine hemorrhage criteria (GCS = 6, hematoma volume 9 mL), the family opted for surgical treatment after informed consent, and the subtemporal transtentorial approach was selected according to the lateral location of the hematoma and the brainstem safe entry zone principles. Emergency surgery was performed via a subtemporal transtentorial approach combined with external ventricular drainage. Under general anesthesia in the supine position, a right-sided external ventricular drain was first placed to facilitate brain relaxation. A preauricular curvilinear incision was then made, a bone flap raised, and the dura opened. The subtemporal corridor was developed, the tentorium incised, and the lateral pontine hematoma meticulously evacuated under microscopic visualization. Postoperative day 1 head CT showed a hematoma clearance rate of approximately 90% with alleviation of brainstem compression. At the 30-day follow-up, the mRS score was 5, consistent with severe disability and a vegetative state. No rebleeding occurred. The patient developed intracranial infection on postoperative day 7, which was successfully managed with antibiotics guided by cerebrospinal fluid culture results (Figure 4).

## 4 Discussion

The management of HPH remains a formidable challenge in neurology and neurosurgery (4). Given the pons’ deep anatomical location and critical functional role, treatment strategy selection demands comprehensive consideration of disease severity, hematoma volume and location, and the patient's overall condition—with all decisions formulated by a multidisciplinary team comprising neurologists, neurosurgeons, and anesthesiologists (31, 35, 36). This approach mitigates biases from individual physician preference, upholds clinical equipoise, and accounts for key influencing factors including anatomical considerations and patient/family treatment preferences.

Our results demonstrate that surgical intervention was associated with significantly better outcomes compared to pharmacological therapy in the 1:1 PSM-matched unbiased cohort. Specifically, the pooled surgical group had a significantly lower 30-day mortality rate (41%) and 90-day mortality rate (50%) compared to the pharmacological therapy group (54% and 63%, respectively). Similarly, the 30-day and 90-day unfavorable outcome rates (mRS ≥ 4) were lower in the surgical group (71% and 77%) than in the pharmacological therapy group (82% and 88%), with the 90-day difference reaching statistical significance (*P* < 0.05) and no significant difference observed for the 30-day outcome (*P* = 0.087). These findings suggest that, under the premise of strictly balanced baseline characteristics via PSM, surgical intervention offers a clear advantage in improving patient survival and long-term functional outcomes. This aligns with and extends previous research. The findings of this study are consistent with the trends reported in previous studies. Specifically, stereotactic puncture and drainage can reduce the 30-day mortality rate by approximately 15% to 30% compared with pharmacological therapy, while also significantly decreasing the rate of unfavorable prognosis by 7.0% to 10 % (12, 14). In addition, Meng et al. reported that the 30-day and 90-day mortality rates of craniotomy for brainstem hemorrhage were 21% and 32%, respectively, which were lower than those in our study. This discrepancy may be attributed to their small sample size of only 19 cases, whereas the rate of unfavorable prognosis (85%) was consistent with our results (16). This indicates that while surgery may not completely prevent unfavorable outcomes in severe HPH, it can significantly reduce mortality risk and improve the chance of survival, even with residual neurological disability, essentially shifting clinical outcomes from death to survivable disability.

Previous investigations into the surgical efficacy for brainstem hemorrhage have either included only a pharmacological therapy group, a solitary craniotomy group, or merely compared pharmacological therapy with stereotactic drainage, and no studies to date have conducted a three-group comparison of pharmacological therapy, stereotactic drainage, and craniotomy (4, 5, 11–14, 16–18, 21, 25, 31). Our study addresses these gaps by providing a head-to-head comparison of stereotactic drainage and craniotomy evacuation within a strictly defined, homogeneous cohort of severe HPH (GCS < 8, hematoma volume ≥ 5 mL, isolated pontine hemorrhage) in the PSM-matched setting. Our findings highlight a critical trade-off in surgical strategy. Craniotomy achieved superior hematoma clearance (90 ± 12% vs. 75 ± 13%, *P* < 0.001), while stereotactic drainage resulted in shorter ICU stays (8 ± 3 vs. 10 ± 3 days, *P* < 0.05), shorter overall hospital stays (19 ± 4 vs. 22 ± 5 days, *P* < 0.001), and lower rates of intracranial infection (5.4% vs. 16%, *P* < 0.05). This suggests a potential paradigm for individualized surgical selection (16, 37, 38). For patients with large, irregular hematomas or signs of active bleeding, where complete evacuation and definitive hemostasis are paramount, craniotomy may be the preferred approach despite its higher invasiveness (4, 31). Conversely, for elderly patients or those with multiple comorbidities who are poor candidates for extensive surgery, stereotactic drainage provides a valuable minimally invasive alternative to reduce mass effect, with the trade-off of potentially lower clearance rates and a need for vigilant monitoring for rebleeding (11, 12, 19, 39).

The integration of preoperative imaging characteristics (hematoma volume, hemorrhage type) and patient-specific factors (age, comorbidities) into a decision-making algorithm warrants further investigation. Notably, our analysis of lower DVT found no significant difference in incidence across all three groups (overall incidence 15%, *P* = 0.271), suggesting that this complication may be related to common factors like prolonged bed rest and reduced mobility rather than the specific treatment modality.

The operational definition of severe HPH applied in this study is specific to adult patients with isolated HPH without severe pre-existing vital organ dysfunction (e.g., acute heart failure, severe liver or renal insufficiency) or coagulation disorders, which is consistent with the inclusion and exclusion criteria of the study. This definition has good clinical applicability in real-world neurological and neurosurgical practice, as it is based on two easily measurable and widely used clinical and imaging indicators (GCS score and hematoma volume) that can be rapidly assessed at the time of patient admission, making it suitable for routine clinical decision-making. However, the generalizability of this definition and the study results is limited in several specific populations: first, it is not applicable to patients with secondary pontine hemorrhage (e.g., caused by cerebrovascular malformations, intracranial aneurysms, or brain tumors) (4, 20), as the pathological mechanism and prognostic factors of secondary pontine hemorrhage are significantly different from those of hypertensive pontine hemorrhage; second, it cannot be extended to patients with severe comorbidities with poor surgical tolerance, as the treatment strategy for such patients is more inclined to pharmacological therapy regardless of hematoma volume; third, it is not suitable for pediatric or adolescent pontine hemorrhage patients, due to the differences in brain development and etiological characteristics. In addition, the study cohort was a single-center population, and the generalizability of the results to multi-center or international populations needs to be verified by further multi-center prospective studies. For clinical centers with similar patient selection criteria and treatment conditions (e.g., availability of stereotactic navigation and microsurgical techniques), the operational definition and study conclusions can provide direct reference for individualized treatment of severe HPH patients.

Acute obstructive hydrocephalus is a common complication of HPH, primarily caused by hematoma compression of the cerebral aqueduct, obstructing cerebrospinal fluid flow, further elevating intracranial pressure, and negatively impacting prognosis (11, 16, 40). In this study, patients with concomitant acute obstructive hydrocephalus who underwent combined external ventricular drainage effectively alleviated hydrocephalus and lowered intracranial pressure, providing a safeguard for hematoma management and neurological recovery. Therefore, for patients with HPH complicated by acute obstructive hydrocephalus, combined external ventricular drainage in addition to hematoma evacuation/drainage is recommended.

Our study also analyzed the length of stay in the ICU and overall hospital stay, which are important indicators reflecting the severity of illness and resource utilization. The pharmacological treatment group had a significantly longer ICU stay (11 ± 3 days) compared with the stereotactic drainage subgroup (8 ± 3 days) and the craniotomy evacuation subgroup (10 ± 3 days). This finding suggests that patients treated pharmacologically may experience a more prolonged period of critical illness requiring intensive monitoring and intervention, which is consistent with their poorer neurological status and higher complication rates. Similarly, the overall hospital stay was longest in the pharmacological treatment group (28 ± 6 days), followed by the craniotomy evacuation subgroup (22 ± 5 days), and shortest in the stereotactic drainage subgroup (19 ± 4 days). The shorter hospital stay in the stereotactic drainage subgroup may be attributed to its minimally invasive nature, which is associated with fewer complications and faster recovery, as reported in previous studies (11, 12, 18). These results highlight the potential benefits of surgical intervention, particularly stereotactic drainage, in reducing healthcare resource consumption and improving patient flow.

Our study identified several independent preoperative prognostic factors for severe HPH using a revised multivariate Logistic regression model that excluded post-treatment variables to avoid bias, based on the PSM-matched cohort. Older age (OR = 1.05, 95% CI: 1.01–1.10, *P* = 0.015), lower admission GCS score (OR = 0.71, 95% CI: 0.62–0.81, *P* < 0.001), larger hematoma volume (OR = 1.13, 95% CI: 1.04–1.22, *P* = 0.004), and presence of acute obstructive hydrocephalus (OR = 3.21, 95% CI: 1.40–7.35, *P* = 0.006) were independent risk factors for 30-day mortality. For 90-day unfavorable outcomes, these factors remained significant, with the addition of massive hematoma morphology as a risk factor (OR = 2.90, 95% CI: 1.32–6.35, *P* = 0.008) and regular antihypertensive use as a protective factor (OR = 0.54, 95% CI: 0.29–0.98, *P* = 0.045). These findings are consistent with previous studies, which have also identified a GCS score ≤ 8, hematoma volume, and presence of acute obstructive hydrocephalus as key prognostic indicators in patients with pontine hemorrhage (16, 41). The protective effect of regular antihypertensive use observed in our study adds to the growing body of evidence highlighting the importance of chronic disease management in improving outcomes for patients with cerebrovascular diseases.

Furthermore, our separate analysis of postoperative variables in surgical patients clarified their role as intermediate outcomes of treatment strategies. Craniotomy evacuation was associated with a significantly higher rate of complete hematoma clearance (≥ 80%) compared to stereotactic drainage (84% vs. 54%, *P* = 0.003). Complete hematoma clearance (≥ 80%) was associated with significantly better 30-day and 90-day outcomes, including lower mortality and unfavorable outcome rates, confirming the importance of achieving adequate hematoma evacuation. This is supported by Meng et al. who reported that greater hematoma clearance was independently associated with better functional recovery in hypertensive pontine hemorrhage patients (16). Conversely, postoperative rebleeding, which occurred exclusively in the stereotactic drainage subgroup (2/37, 5.4%), was a strong predictor of poor prognosis, with 100% 30-day mortality and 90-day unfavorable outcome rates in affected patients. This highlights the need for careful patient selection and strict perioperative hemostatic management in stereotactic drainage procedures to minimize this devastating complication.

By distinguishing between preoperative prognostic determinants for clinical decision-making and postoperative intermediate endpoints, our study avoids post-treatment bias and ensures the scientific rigor of our findings. The identification of modifiable factors, such as regular antihypertensive use and complete hematoma clearance, provides potential targets for improving outcomes in patients with severe HPH. Future studies should validate these findings in larger cohorts and explore interventions to optimize these factors.

It is crucial to emphasize that HPH, as a severe subtype of brainstem hemorrhage, carries an extremely poor overall prognosis (3, 22, 23). Even with standardized treatment, the severe patients (preoperative GCS < 8) included in this study still face a high probability of long-term adverse outcomes such as persistent coma, vegetative state, or lifelong dependency on care. This imposes a heavy medical and economic burden on patients’ families and society, a problem particularly acute in regions with limited medical resources.

Therefore, while this study confirms some efficacy advantages of surgery over pharmacological therapy and provides comparative data between two major surgical approaches, it does not advocate for aggressive surgical intervention in all cases of HPH. Clinical decisions still require individualized consideration, balancing patient condition, available medical resources, and family expectations. Future prospective studies with larger sample sizes and longer follow-up are needed to further validate the efficacy, safety, and long-term prognosis of different treatment strategies, and to refine patient selection criteria for each surgical approach.

This study has several limitations: ① As a retrospective study, despite strict control over key baseline factors like preoperative GCS and hematoma volume, selection bias may still exist. Non-randomized treatment assignment was mainly determined by the informed choice of patients’ families, which may introduce unmeasured confounding factors related to socioeconomic status and family attitude. ② The sample size is relatively limited, especially for the craniotomy subgroup and patients with hydrocephalus, potentially affecting the reliability of some statistical findings. ③ All data were collected from a single tertiary medical center, which may restrict the generalizability of the conclusions to other clinical settings. ④ The 90-day follow-up duration may be insufficient to reflect the full potential of neurological recovery, as long-term functional improvement often continues beyond 3 months post-injury. ⑤ Although we adopted PSM and IPW to balance baseline characteristics and reduce confounding by indication, 1:1 PSM inevitably reduced the effective sample size and statistical power. Furthermore, even after rigorous statistical adjustment, residual unknown confounding could not be completely eliminated due to the observational non-randomized nature of the study. All these limitations are fully acknowledged and should be considered when interpreting the study results.

## 5 Conclusion

This study conducted all core analyses solely on a 112-patient 1:1 PSM-matched cohort of patients with severe HPH, with the aim of reducing the potential for confounding bias related to surgeon and family treatment choice decisions. Within this matched cohort, we observed that surgical intervention was correlated with significantly lower 30/90-day mortality and reduced risk of 90-day unfavorable functional outcomes, with stereotactic drainage and craniotomy showing divergent safety and efficacy characteristics. The findings of this study do not constitute a formal treatment recommendation, but may provide reference for individualized clinical decision-making when combined with patient-specific and hematoma-related factors. It should be emphasized that this is a retrospective single-center study, which cannot establish a definitive causal relationship between treatment and outcomes, and the results should be interpreted with caution; further large-scale multi-center prospective studies in unbiased patient populations are needed to verify the robustness of the findings.

## Data Availability

The raw data supporting the conclusions of this article will be made available by the authors, without undue reservation.
